# Outcome and critical care resources utilised by do not attempt cardiopulmonary resuscitation (DNACPR) patients admitted to the ICU at a tertiary hospital in Saudi Arabia: a retrospective review of the critical care database

**DOI:** 10.1186/s13054-025-05656-5

**Published:** 2025-09-29

**Authors:** Asiah Rugaan, Muath Mobarki, Soltan Mohammad Hamida, Masood Iqbal, Manar Alotibi, Tafe Abdulelah Howsawi, Sulafah Reda, Hanan Abdullah Alzhrani, Asmaa Saeed Almadani, Adeel Ahmed Khan

**Affiliations:** 1 King Abdullah Medical City, Makkah, Kingdom of Saudi Arabia; 2 Saudi Board Program of Preventive Medicine, Makkah Healthcare Cluster, Makkah, Kingdom of Saudi Arabia

**Keywords:** Critical care resource utilization, Do not attempt resuscitation (DNAR), Do not attempt cardiopulmonary resuscitation (DNACPR), Intensive care unit (ICU), End-of-life care, Terminal illness, Palliative care, Ethical decision-making

## Abstract

**Background:**

The Do Not Attempt Cardiopulmonary Resuscitation (DNACPR) order. aims to prevent the initiation of inappropriate, aggressive interventions in patients with a poor prognosis, highlighting the need to assess intensive care unit (ICU) resource utilization in such cases. Therefore, our study aimed to evaluate the resources utilized by DNACPR patients and compare them with those utilized by non-DNACPR patients in the intensive care unit to explore the outcomes of these patients.

**Methods:**

A retrospective cohort study of 7104 patients admitted to the ICU in King Abdullah Medical City, Makkah, Saudi Arabia, was performed. Patients were segregated into DNACPR cases and non-DNACPR cases. Data were extracted from the critical care registry from January 2016 to June 2023. A descriptive analysis was performed. Multivariate analysis was used to adjust for the severity of illness between groups and compare outcomes for resources utilized by the study population after the DNACPR decision was made, between DNACPR patients and non-DNACPR patients.

**Results:**

Over eight years, a total of 7,104 patients were admitted to the ICU, with 988 classified as DNACPR (13.9%) and 6,116 (86.1%) classified as non-DNACPR patients. DNACPR patients utilized a substantial amount of critical care resources, including mechanical ventilation (88.9% vs. 41.4%, AOR 7.8, 95% CI (6.1–9.9), *P* < 0.001) and continuous renal replacement therapy (CRRT) (28.6% vs. 6.7%, AOR 4.4, 95% CI (3.6–5.4), *p* < 0.001). All radiological imaging was significantly utilized by DNACPR versus non-DNACPR patients (*P* < 0.001). Additionally, blood product transfusions were significantly consumed by DNACPR versus non-DNACPR patients (*P* < 0.001). On the other hand, the mortality rate for DNACPR patients was markedly higher (76.7%) than that for non-DNACPR patients (7.7%) (*P* < 0.0001). The mean ICU length of stay for DNACPR patients was 20.4 days, whereas it was 8.0 days for non-DNACPR patients (*P* < 0.001). In subgroup analysis of only emergent admissions, the utilization of ICU interventions, such as mechanical ventilation, CRRT, radiological imaging, and blood transfusion, was significantly higher among DNACPR patients versus non-DNACPR patients, with *P* < 0.001.

**Conclusion:**

DNACPR patients consumed a significant amount of ICU resources after the DNACPR decision was made. The findings underscore significant disparities in both resource consumption and clinical outcomes, highlighting the need for optimized care strategies for terminally ill patients in the ICU setting.

## Background

Do Not Attempt Cardiopulmonary Resuscitation (DNACPR) directives are a broad, often ambiguously used term in medical practice worldwide [1, 2]. Its primary intent is to prevent medically inappropriate interventions and patient harm when aggressive medical treatments are no longer expected to alter a patient’s prognosis [3]. However, the scope of “DNACPR” directives varies significantly, ranging from a narrow focus on Do Not Attempt Cardiopulmonary Resuscitation (DNACPR); specifically addressing cardiac or respiratory arrest to a broader Do Not Attempt Resuscitation (DNAR) that can encompass withholding various life-sustaining measures, such as Intensive Care Unit (ICU) admission, mechanical ventilation, and vasopressors. This distinction is crucial, as patients with a DNACPR order may still continue to receive other forms of resuscitative organ support (e.g., ventilation, renal replacement therapy). In many countries, DNACPR decisions vary and are influenced by range of ethical, legal, and cultural factors [1, 2, 4]. Similarly, in Saudi Arabia, the implementation of DNACPR decisions is variable due to the same factors [5, 6].

In Saudi Arabia, the concept of DNAR (referring broadly to the principle of not attempting resuscitation) was approved by an Islamic law (Fatwa issued in 1989, Decree No. 12086) and further endorsed by the Ministry of Health’s (MOH) national policy in 2017 [7–9]. The DNAR decision is made for advanced terminally ill patients when the medical outcome is deemed dismal [8–10]. These regulations authorize the cessation of aggressive treatment once three specialized consultants reach a consensus for the DNAR order. Subsequently, the patient’s management should be diverted towards palliative care goals rather than intensive care treatment [10, 11].

Although both the Islamic law and the MOH policy permit withholding as well as withdrawing aggressive measures, for preventing harm, and prolonging the suffering of terminally ill patients with incurable illness. However, in practice, this is limited to withholding cardiopulmonary resuscitation when cardiac arrest occurs, while all medical interventions are continued till the late stage of illness [12, 13].

The implementation of DNAR policy in healthcare settings in Saudi Arabia is variable due to inconsistencies in practice among physicians, family pressure, and potential cultural restraints [9–14]. In this context, patients for whom a DNAR order has been made in intensive care units often continue to receive aggressive measures where the risks outweigh the benefits, leading to high mortality and poor quality outcomes [3, 14–18]. This suggests that in practice, what is often implemented is a DNACPR order, allowing for continued organ support while Cardiopulmonary Resuscitation (CPR) is withheld.

This study focused on DNACPR orders, recognizing that while these decisions intersect with ethical considerations and end-of-life care goals [19], their precise influence on ICU resource utilization, particularly when other organ support continues, requires further exploration.

Prior studies indicate that patients with DNACPR orders may still receive aggressive interventions and often occupy ICU beds for extended durations, potentially limiting access for patients with better prognoses [19]. Additionally, delays in enacting DNACPR decisions are associated with increasing use of interventions such as mechanical ventilation and vasopressors, as well as longer ICU stays (Khandelwal et al.) [20]. A retrospective analysis conducted in surgical ICUs revealed that early decision-making was associated with decreased resource use and healthcare costs (Prendergas& Luce) [21].

The cost of intensive care services is high compared to other hospital resources, and the demand for intensive care beds is increasing worldwide due to advances in medicine and the provision of highly specialized services [18, 22–25]. Early patient selection for ICU admissions will facilitate the limitation of intensive care resources, necessitating the proper utilization of ICU services for managing patients with treatable illnesses or when the outcome is expected to result in a reasonable quality of life [18, 26, 27]. Conversely, the care of patients with DNACPR orders, who are not expected to benefit from aggressive life-sustaining measures, could be reallocated to hospice or palliative care services instead [22, 28–30].

Despite the importance of this topic, limited research has examined the ICU resource consumption among patients with DNACPR orders when there is improper selection of ICU admission or a delay in the end-of-life care plan. The objective of this study is to address this gap by exploring the use of critical care resources, such as ICU bed days, mechanical ventilation, dialysis, radiologic imaging, and other interventions, among DNACPR patients and the outcome of these patients. The ultimate goal of the study is to promote efficient, patient-centered use of critical care services.

## Methods

### Study design, participants and setting

This retrospective cohort study was conducted on patients admitted to the intensive care department at King Abdullah Medical City (KAMC), Makkah, Saudi Arabia, between January 1, 2016, and January 6, 2023. Patients were segregated into DNACPR cases and non- DNACPR cases. KAMC is a tertiary-care facility with a capacity of over 500 beds. The ICU service at KAMC follows a closed system model and is staffed by board-certified intensivists providing round-the-clock coverage, 24 h a day, 7 days a week. Daily multidisciplinary rounds were conducted, and families were regularly updated and engaged in patient care planning [23]. Patients included in this study were those classified as having DNACPR after ICU admission. The study received approval from the Institutional Review Board (IRB- 21–865).

### Sample size

The total population of patients included 7104 patients admitted to the ICU during the study period from January 1, 2016, to January 6, 2023. The DNACPR patients numbered 988, whereas the non-DNACPR patients numbered 6116.

#### Inclusion criteria

All adult patients with signed DNACPR document were included in the study. The DNACPR decision made based on the consensus agreement of three consultants, including the admitting primary specialty consultant and two intensive care consultants. Patients decided to be DNACPR were those had untreatable advanced terminal illness, or sever multiorgan failure refractory to medical therapy, in whom death is imminent and resuscitation would not change the prognosis. At KAMC, the DNAR order indicates that the patient shall continue to have all resuscitative measures that have been initiated, but once cardiac or respiratory arrest happens, no CPR shall be attempted (DNACPR); there is no withdrawal of life-sustaining therapies once initiated. Furthermore, all medical interventions for patient medical care will be continued based on physician’s decision. The mechanism of death among the DNACPR group was due to cardiorespiratory arrest, which occurred without any withdrawal of life-sustaining treatment.

Patients meeting any of the following criteria were excluded: (1) absence of documented DNACPR data in patient records, (2) age younger than 18 years, or (3) incomplete or insufficient medical records for analysis. (4) DNACPR was performed after discharge from the ICU. (5) Patients whose DNACPR decision was reversed. As a result, 104 patients were excluded.

The data for this study were collected in an Excel sheet through a retrospective review of prospectively collected data in the critical care database registry, and missing data were obtained from an open chart review. Then, the data were extracted into Statistical Package for Social Sciences (SPSS) version 26 for analysis.

### Study tool

The critical care database registry is IRB-approved as of 12 January 2014 (IRB No. 14–155). A secure password protects the database registry, was accessible only to authorized research coordinators. The database registry is designed to capture data on ICU patients, including demographics and a range of ICU resource variables, such as the length of ICU stay, specific interventions or procedures, antibiotics administered, blood products received, and imaging studies ordered. The patient demographics included (age, gender), comorbidities, severity of illness scores (APACHE IV, SOFA), primary diagnosis for ICU admission, and advance directives were recorded. Outcomes such as ICU and hospital mortality rates were also evaluated. In this study, the resources used for DNACPR patients included only those utilized after the DNACPR decision was made during ICU admission. In contrast, resources used for non-DNACPR patients were recorded throughout the entire ICU stay.

### Data analysis

The data analyses were carried out using SPSS version 26 (Armonk, New York: IBM Corporation). Descriptive statistics were used to summarize the data, with categorical variables presented as frequencies and percentages and continuous variables expressed as the means ± standard deviations (SDs) or medians with interquartile ranges (IQRs), depending on the distribution. Comparisons between DNACPR patients and non- DNACPR patients were made using Pearson’s chi-square test for categorical variables, whereas Student’s t test and the Mann‒Whitney U test were used for continuous variables depending on the distribution. Normality tests were performed via the Kolmogorov–Smirnov and Shapiro–Wilk tests. Subgroup analysis for emergency admissions has been conducted, excluding all elective admissions, as most admissions fall into the non-DNACPR group. Multivariate logistic regression analysis has been done for adjustment for the differences in severity of illness between the groups controlling for APACHI IV and cancer comorbidity. A p-value of < 0.05 was considered statistically significant.

## Results

The characteristics of the ICU patients (*n* = 7,104) are summarized in Table 1. During the study period, 7,104 patients were admitted to the intensive care unit (ICU). Among them, 988 (13.9%) patients had a documented Do Not Attempt Cardio Pulmonary Resuscitation (DNACPR) order after ICU admission, while 6,116 (86.1%) patients were classified as non-DNACPR (full code).

**Table 1:** Characteristics of do not attempt cardiopulmonary resuscitation (DNACPR) patients and non-DNACPR patients admitted to the ICU of King Abdullah medical City (KAMC) from 2016–2023 (*n* = 7104)

Variable		DNACPR (*n* = 988)			Non DNACPR (*n* = 6116)	*P*-value	
**Age** ^**a**^							
Mean ± SD		61.4 ± 16.2			55.9 ± 16.6	**< 0.001**	
**Gender**							
Male		544 (55.1%)			3242 (53.0%)	0.230	
Female		444 (44.9%)			2874 (47.0%)		
**Smoking Status**							
Smoker		306 (31.0%)			1657 (27.1%)		
Non smoker		545 (55.2%)			3239 (53.0%)	**< 0.001**	
Unknown		137 (13.9%)			1220 (19.9%)		
**Source of admission**							
ER		383 (38.8%)			2173 (35.5%)		
OR		88 (8.9%)			2081 (34.0%)	**< 0.001**	
Inpatient		517 (52.3%)			1862 (30.4%)		
**Comorbidity**							
None		27 (2.7%)			400 (6.5%)	**<0.001**	
Hypertension		548 (55.5%)			3308 (54.1%)	0.420	
Diabetes		491 (49.7%)			2783 (45.5%)	**0.014**	
Chronic cardiac		351 (35.5%)			1865 (30.5%)	**0.002**	
Chronic renal failure		208 (21.1%)			1008 (16.5%)	**<0.001**	
Chronic lung disease		85 (8.6%)			555 (9.1%)	0.631	
Hypothyroidism		69 (7.0%)			414 (6.8%)	0.804	
Chronic liver disease		69 (7.0%)			154 (2.5%)	**<0.001**	
Autoimmune disease		9 (0.9%)			75 (1.2%)	0.395	
Cancer		419 (42.4%)			2137 (34.9%)	**<0.001**	
Malignancy hematology		97 (9.8%)			321 (5.2%)	**<0.001**	
Malignancy non-hematology		325 (32.9%)			1829 (29.9%)	**0.058**	
Bed ridden		174 (17.6%)			694 (11.3%)	**<0.001**	
**Reason of admission **							
Septic shock		658 (66.6%)			**2043 (33.4%)**	**<0.001**	
Respiratory failure		521 (52.7%)			1813 (29.6%)	**<0.001**	
Postoperative/post Procedure		78 (7.9%)			2106 (34.4%)	**<0.001**	
Neurological diseases and disorders		298 (30.2%)			1624 (26.6%)	**0** **.018**	
Cardio vascular emergencies		39 (3.9%)			465 (7.6%)	**<0.001**	
Post RRT		112 (11.3%)			325 (5.3%)	**<0.001**	
Uncontrolled bleeding		62 (6.3%)			266 (4.3%)	**0.007**	
Electrolyte or acid base disturbance		123 (12.4%)			457 (7.5%)	**<0.001**	
Metabolic Disorder		65 (6.6%)			247 (4.0%)	**<0.001**	
Post CPR		99 (10.0%)			100 (1.6%)	**<0.001**	
Shock Stat		444 (44.9%)			829 (13.6%)	**<0.001**	
**ICU scores**							
**APACHI 4** ^**a**^		(n=799)			(n=4773)		
Mean ±SD		57.4 ± 34.5			39.1 ± 26.4	**<0.001**	
**SOFA** ^**a**^		(n=979)			(n=5548)		
Mean ±SD		7.5 ± 4.9			4.4 ± 4.2	**<0.001**	
**Time from ICU admission to DNACPR decision**							
Mean ±SD		11.91±17.63			______		
**Time from DNACPR decision to discharge**							
Mean ±SD		9.21±16.2			______		
**Number of DNACPR patients died within 24 hours from DNACPR decision**		210 (21.3%)					

^a^ P value was calculated via the Mann–Whitney U test, bold values represent significant p-value <0.05

The DNACPR patients were significantly older than the non- DNACPR patients, with a mean age of 61.4 ± 16.2 years versus 55.9 ± 16.6 years, respectively (*p* < 0.001). Approximately 31% (*n* = 306) of DNACPR patients and 27% (*n* = 1,657) of non-DNACPR patients were smokers. DNACPR patients were admitted more frequently from inpatient departments than non- DNACPR patients (52.4% vs. 30.4%, *p* < 0.001). At admission, the APACHE IV and SOFA scores were significantly higher among DNACPR patients (57.4 ± 34.5 vs. 39.1 ± 26.4, *p* < 0.001 and 7.5 ± 4.9 vs. 4.4 ± 4.2, *p* < 0.001, respectively). Several underlying comorbidities were significantly more prevalent among DNACPR patients than non- DNACPR patients, including diabetes (49.7% vs. 45.5%, *p* = 0.014), cardiac diseases (35.5% vs. 30.5%, *p* = 0.002), renal failure (21.2% vs. 16.5%, *p* < 0.001), chronic liver disease (7.0% vs. 2.5%, *p* < 0.001), and malignancy (42.4% vs. 34.9%, *p* < 0.001). Additionally, DNACPR patients were significantly more likely to be bedridden compared to non-DNACPR patients (17.6% vs. 11.3%, *p* < 0.001). The most common reasons for ICU admission among DNACPR patients were sepsis (66.6% vs. 34%, *p* < 0.001), respiratory failure (52.7% vs. 29.6%, *p* < 0.001), and hemodynamic instability (44.9% vs. 13.6%, *p* < 0.001). In contrast, the most common reason for ICU admission among non- DNACPR patients was postoperative care following major elective surgery (34.4% vs. 7.9%, *p* < 0.001).

### DNACPR group results

Concerning the DNACPR patients, the time from ICU admission to DNACPR order was (mean 11.9 ± 17.6), while the time from DNACPR order to death (mean 9.2 ± 16.2), and the number of DNACPR patients died within 24 h from DNACPR order were 210 (21%) from total DNACPR patients. The reasons of DNACPR has been identified among 620 (63%) of DNACPR patients (Table 1). The commonest reasons of DNACPR as following: terminal Illness advanced cancer in 247 (39.8%) patients, multiple sever co-morbidities with poor quality of life (bed ridden) in 148 (23.8%), severe neurological impairment with poor prognosis for recovery in 110 (17.7%), and refractory shock multi-organ failure in 78 (12.6%) (Table 2). All patients signed as DNACPR were on full support of care with exception of minor number of case whom had new intervention after DNACPR order. After DNACPR order; new mechanical ventilation were given to among 28 patients and new CRRT started on 14 patients only.

**Table 2:** DNACPR reasons for patients admitted at ICU of King Abdullah medical City (KAMC) during 2016–2023 (*n* = 620)

DNACPR Reasons	(*n* = 620) No. %
Terminal illness, advanced cancer, not candidate for active cancer therapy	247 (39.8%)
Multiple sever co-morbidities with poor quality of life (Bed ridden)	148 (23.8%)
Sever neurological impairment Poor prognosis for recovery	110 (17.7%)
Refractory Septic shock multi-organs failure	78 (12.6%)
End stage liver failure with refractory septic shock with multi-organ failure	21 (3.39%)
End stage lung disease-not transplant candidate mechanical ventilator dependent	11 (1.77%)
End stage cardiomyopathy-not transplant candidate poor quality of life (Bed ridden)	5 (0.8%)

### ICU resource comparing DNACPR vs. non- DNACPR results

DNACPR patients utilized resources significantly more than non- DNACPR patients (Table 3). CRRT utilization was significantly higher among DNACPR patients (283 [28.6%] versus 412 [6.7%]; *P* < 0.001). Mechanical ventilation utilization was significantly higher among DNACPR patients (878 [88.9%] versus 2530 [41.4%]; *P* < 0.001). The endoscopy procedure was performed for 55 (5.6%) DNACPR patients and 115 (1.9%) DNACPR patients (*P* < 0.001). Sedation was administered to 843 (85.3%) DNACPR patients and 2141 (35.0%) non- DNACPR patients; *P* < 0.001.

**Table 3:** Comparison of ICU resources utilized for DNACPR patients and non-DNACPR patients admitted to the ICU of King Abdullah medical City (KAMC) from 2016–2023 (*n* = 7104)

Variable			DNACPR (*n* = 988)	Non DNACPR (*n* = 6116)	*P*-value	
**Dialysis (CRRT)**			283 (28.6%)	412 (6.7%)	**< 0.001**	
Number of CRRT			295	421		
Median (IQR)^a^			1 (1–1)	1 (1–1)	0.279	
**Mechanical ventilation**			878 (88.9%)	2530 (41.4%)	**<0.001**	
Invasive ventilation			843 (85.3%)	2141 (35.0%)	**<0.001**	
Non-invasive ventilation			35 (3.5%)	389 (6.4%)	**<0.001**	
Tracheostomy			254 (30%)	381(17.7%)	**<0.001**	
Mechanical ventilation duration in days			17,579	14000	**<0.001**	
Median (IQR)^a^			11 (4-24)	3 (1-9)	**<0.001**	
**Endoscopy**			55 (5.6%)	115 (1.9%)	**<0.001**	
**Received Sedation**			843 (85.3%)	2141 (35.0%)	**<0.001**	
**Imaging radiology**						
**CT**			590 (59.7%)	3268 (53.4%)	**<0.001**	
CT Number			1865	8099		
Median (IQR)^a^			3 (2-4)	2 (1-3)	**<0.001**	
**MRI**			280 (28.3%)	1203 (19.7%)	**<0.001**	
MRI Number of studies			477	1779		
Median (IQR)^a^			1 (1-2)	1 (1-2)	**< 0.001**	
**Ultrasound**			298 (30.2%)	972 (15.9%)	**<0.001**	
Ultrasound Number of studies			373	1166		
Median (IQR)^a^			1 (1-1)	1 (1-1)	0.110	
**Intervention radiology**			206 (20.9%)	871 (14.2%)	**<0.001**	
Intervention Number of studies			342	1216		
Median (IQR)^a^			1 (1-2)	1 (1-2)	**<0.001**	
**ECO**			212 (21.5%)	641 (10.5%)	**<0.001**	
ECO Number of studies			237	693		
Median (IQR)^a^			1 (1-1)	1 (1-1)	0.108	
**Antibiotics use**			849 (85.9%)	4619 (75.5%)	**<0.001**	
One antibiotic			202 (23.9%)	1988 (43.2%)	**<0.001**	
Two or more antibiotic			642 (76.1%)	2610 (56.8%)		
Antibacterial			1835	8379		
Antiviral			107	515		
Antifungal			38	67		
**Blood Transfusion **						
**PRBCs**			149 (15.1%)	253 (4.1%)	**<0.001**	
Number PRBCs of Units			352	551		
**FFP**			83 (8.4%)	114 (1.9%)	**<0.001**	
Number of FFP Units			390	526		
**Platelets**			72 (7.3%)	82 (1.3%)	**<0.001**	
Number of Platelets Units			640	758		
**Cryoprecipitate**			5 (0.5%)	7 (0.1%)	**0.005**	
Number of Cryoprecipitate Units			6	26		

^a^ P value was calculated via the Mann–Whitney U test, bold values represent significant p-value <0.05

With respect to imaging studies and procedures, approximately 590 (59.7%) DNACPR patients underwent CT imaging, whereas 3268 (53.4%) non-DNACPR patients underwent CT imaging (*P* < 0.001). The median number of CT scans was 3 (2–4 IQR) for DNACPR patients and 2 (1–3 IQR) for non- DNACPR patients. On the other hand, 280 (28.3%) DNACPR patients underwent MRI, and 1203 (19.7%) non- DNACPR patients underwent MRI (*P* < 0.001). Ultrasound imaging was performed for 298 (30.2%) DNACPR patients and 972 (15.9%) non-DNACPR patients **(***P* < 0.001). The number of interventional radiology procedures was significantly higher among DNACPR patients (206 (20.9%) versus 871 (14.2%), *P* < 0.001). In addition, echocardiography imaging (ECHO**)** was significantly more common for DNACPR patients (212 (21.5%) versus 641 (10.5%), *P* < 0.001).

Regarding antimicrobial therapy utilization, DNACPR patients tended to receive significantly more antimicrobial combinations, with 76.1% of DNACPR patients receiving two or more antibiotics, compared to 56.8% of non-DNACPR patients.

In addition, the utilization of blood and blood products was significantly higher among DNACPR patients. Approximately 149 (15.1%) DNACPR patients received 352 units of PRBCs, whereas 253 (4.1%) non-DNACPR patients received 551 units (*P* < 0.001). Eighty-three (8.4%) DNACPR patients received 390 units of FFP, whereas 114 (1.9%) non-DNACPR patients received 526 units of FFP (*P* < 0.001). Approximately 72 (7.3%) DNACPR patients received 640 units of platelets, whereas 82 (1.3%) non-DNACPR patients received 758 units of platelets (*P* < 0.001). The average length of stay for DNACPR patients was significantly higher compared to non-DNACPR patients (20.4 ± 25.1 versus 8.0 ± 11.2 (*P* < 0.001)). Additionally, the mortality rate was significantly higher (76.7%) among DNACPR patients compared to non-DNACPR patients (7.7%) (*P* < 0.001).

### Multivariate logistic regression

Table 4 presents multivariate logistic regression analysis after adjustment between the groups for severity of illness after controlling for APACHI IV score and cancer as a comorbidity. The analysis showed that DNACPR patients are significantly receiving, CRRT (AOR 4.4, 95% CI 3.6–5.4), mechanical ventilation (AOR 7.8, 95% CI 6.1–9.9), sedations (AOR 7.5, 95% CI 6.0-9.3), MRI (AOR 1.3, 95% CI 1.1–1.5), ultrasound (AOR 1.7, 95% CI 1.4–2.1), intervention radiology (AOR 1.4, 95% CI 1.6–1.7), ECHO (AOR 2.2, 95% CI 1.8–2.8), endoscopy (AOR 2.1, 95% CI 1.4–3.3), and blood transfusion (AOR 2.9, 95% CI 2.2–3.8) with significant *P* > 0.001 respectively. However, the utilization of CT and antibiotics was not significantly different between the two groups after adjustment for the differences in severity of illness and cancer as a comorbidity.

**Table 4:** Multivariate logistic regression for resources consumed by DNACPR patients after adjustment for severity of illness between groups (APACHI IV and cancer comorbidity) (*n* = 7104)

Intervention	Adjusted Odds Ratio	95% Confidence Interval	*P* value
Mechanical ventilation	7.82	(6.13, 9.96)	0.001
Sedation	7.55	(6.06, 9.39)	0.001
CRRT	4.47	(3.64, 5.48)	0.001
Tracheostomy	3.98	(3.22, 4.93)	0.001
Ultrasound	1.75	(1.45, 2.11)	0.001
CT	1.16	(0.99, 1.36)	0.062
MRI	1.32	(1.10, 1.59)	0.003
Intervention radiology	1.42	(1.16, 1.74)	0.001
ECHO	2.28	(1.85, 2.82)	0.001
Endoscopy	2.19	(1.41, 3.39)	0.001
PRBCs transfusion	2.91	(2.21, 3.84)	0.001
Platelet transfusion	3.27	(2.15, 4.96)	0.001
Fresh frozen transfusion	2.99	(2.06, 4.33)	0.001
Antibiotic	1.03	(0.83, 1.28)	0.788

### Sub-group analysis of emergent admissions (excluding elective planned admissions due to postoperative surgery) comparing DNACPR with non-DNACPR patients (Tables 5, 6, 7, 8 and 9)

A total of 2184 planned admissions were excluded from the analysis to study the emergency admissions subgroup, 2106 patients among the non-DNACPR, while 78 patients among the DNACPR were excluded (Table 5). Comparing DNACPR with non-DNACPR patients’ emergency admissions. DNACPR patients’ emergency admissions were significantly older (mean ± SD: 61.47 ± 16.2 years) compared to non- Comparing DNACPR with non-DNACPR patients (57.10 ± 16.8 years) (*P* < 0.001). The DNACPR patients had a higher prevalence of Chronic liver disease (7.5% vs. 2.9%, *P* < 0.001), Cancer (41.5% vs. 23.2%, *P* < 0.001), and Bedridden status (10.1% vs. 6.5%, *P* < 0.001). Conversely, hypertension was more prevalent among non-DNACPR (56.3% vs. 60.4%, *P* = 0.020), while all other comorbidities were comparable between the 2 groups. The most common reasons for admission in both groups were septic shock and respiratory failure; however, DNACPR patients had a significantly higher rate of admissions with septic shock and respiratory failure compared to non-DNACPR patients (68.9% vs. 47.2%, *P* < 0.001, and 56.6% vs. 43.7%, *P* < 0.001, respectively). In contrast, non-DNACPR had significantly higher admissions with neurological and cardiovascular emergencies (31.1% vs. 34.9%, *P* = 0.031, 4.1% vs. 9.9%, *P* < 0.001, respectively). Post-CPR (10.4% vs. 2.3%, *P* < 0.001). Post RRT (12.3% vs. 7.8%, *P* < 0.001). The calculated APACHE IV and SOFA scores upon ICU admission were significantly higher among the DNACPR group (mean ± SD: 68.96 ± 30.53 vs. 46.94 ± 26.518, *P* < 0.001, and mean ± SD: 7.48 ± 4.8 vs. 4.68 ± 3.84, *P* < 0.001, respectively). Indicating from the time of admission to ICU, DNACPR patients were more severely ill and with more organ failure than the non-DNACPR group, and a poor outcome is expected from the time of admission. Although all the interventions measured among DNACPR group were collected after DNACPR order date, and despite the multivariate regression adjustment for the differences between groups with regards to severity of illness (APACHI IV), organ dysfunction (SOFA) and cancer comorbidity. the DNACPR group showed a significantly higher utilization of various critical care interventions compared to non- DNACPR group (Table 9), including Mechanical ventilation days: AOR = 4.9 (95% CI: 4.77–5.06, *P* = 0.0001), CRRT days: AOR = 2.46 (95% CI: 2.04–2.96, *P* = 0.0001), Ultrasound: AOR = 1.20 (95% CI: 1.03–1.35, *P* = 0.021), CT: AOR = 1.13 (95% CI: 1.06–1.21, *P* = 0.001), MRI: AOR = 1.18 (95% CI: 1.03–1.35, *P* = 0.014), Intervention radiology: AOR = 1.27 (95% CI: 1.09–1.50, *P* = 0.003), ECHO: AOR = 1.98 (95% CI: 1.58–2.49, *P* = 0.0001), and Blood Transfusions (PRBCs, Platelets, FFP): All showed significantly higher odds for the number of units including: PRBCs: AOR = 2.35 (95% CI: 1.94–2.85, *P* = 0.0001), Platelets: AOR = 1.7 (95% CI: 1.47–1.98, *P* = 0.0001), Fresh frozen transfusion: AOR = 2.31 (95% CI: 1.90–2.80, *P* = 0.0001). with regards to length of stay and mortality outcomes (Table 7). The mean ICU LOS for DNACPR patients was 20.5 ± 25.3 days, significantly longer than for non-DNACPR patients (9.26 ± 11.9 days) (*P* < 0.001). the DNACPR patients had a substantially higher ICU mortality rate (77.3% vs. 10.8%, *P* < 0.001).

**Table 5:** Sub-group analysis characteristic of do not attempt cardiopulmonary resuscitation (DNACPR) patients and non-DNACPR patients for emergency ICU admission (excluding planned elective admissions for postoperative monitoring)

Variable	DNACPR (*n* = 910)	Non DNACPR (*n* = 4010)	*P*-value
**Age**			
Mean ± SD	61.47 ± 16.2	57.10 ± 16.8	**< 0.001**
**Gender**			
Male	506 (55.6%)	2082 (51.9%)	0.044
Female	404 (44.4%)	1928 (48.1%)	
**Smoking Status**			
Smoker	287 (36.7%)	1124 (34.5%)	0.228
Non smoker	494 (63.3%)	2138 (65.5%)	
**Comorbidity**			
Hypertension	512 (56.3%)	2424 (60.4%)	**0.020**
Diabetes	454 (49.9%)	2060 (51.4%)	0.420
Chronic cardiac disease	330 (36.3%)	1445 (36.0%)	0.897
Chronic renal failure	196 (21.5%)	891 (22.2%)	0.655
Chronic lung disease	80 (8.8%)	427 (10.6%)	0.096
Hypothyroidism	65 (7.1%)	308 (7.7%)	0.580
Chronic liver disease	68 (7.5%)	118 (2.9%)	**< 0.001**
Autoimmune disease	8 (0.9%)	62 (1.5%)	0.125
Cancer	378 (41.5%)	931 (23.2%)	**< 0.001**
Malignancy hematology Malignancy	94 (10.3%)	288 (7.2%)	**< 0.001**
Non-hematology	287 (31.5%)	649 (16.2%)	**< 0.001**
Bed ridden	92 (10.1%)	261 (6.5%)	**< 0.001**
**Reason of admission**			
Septic shock	627 (68.9%)	1893 (47.2%)	**<0.001**
Respiratory failure	515 (56.6%)	1752 (43.7%)	**<0.001**
Neurological diseases and disorders	283 (31.1%)	1398 (34.9%)	**0.031**
Cardio vascular emergencies	37 (4.1%)	395 (9.9%)	**<0.001**
Post RRT	112 (12.3%)	314 (7.8%)	**<0.001**
Uncontrolled bleeding	61 (6.7%)	239 (6.0%)	0.398
Electrolyte or acid base disturbance	121 (13.3%)	439 (10.9%)	0.044
Metabolic Disorder	65 (7.1%)	238 (5.9%)	0.171
Post CPR	95 (10.4%)	92 (2.3%)	**<0.001**
Shock Stat	427 (46.9%)	783 (19.5%)	**<0.001**
**ICU scores**			
**APACHI 4** ^**a**^	(n=739)	(n=3125)	
Mean ± SD	68.96 ± 30.53	46.94 ± 26.518	**<0.001**
**SOFA** ^**a**^	(n=907)	(n=3987)	
Mean ± SD	7.48 ± 4.8	4.68 ± 3.84	**<0.001**

^a ^P-value has been calculated using Mann Whiney U-test, CPR: Cardio-Pulmonary Resuscitation, RRT: Rapid Response Team, bold values represent significant p-value <0.05

**Table 6:** Sub-group analysis comparison of ICU resources utilized for DNACPR patients and non-DNACPR patients for emergency ICU admission (excluding planned elective admissions for postoperative monitoring)

Variable	DNACPR (*n* = 910)	Non DNACPR (*n* = 4010)	*P*-value
**Dialysis (CRRT)**	272 (29.9%)	374 (9.3%)	**< 0.001**
Number of CRRT	284	382	
Median (IQR)^a^	1 (1–1)	1 (1–1)	0.237
**Mechanical ventilation**	807 (88.7%)	1757 (43.8%)	**<0.001**
Invasive ventilation	772 (95.7%)	1382 (78.7%)	**<0.001**
Non-invasive ventilation	35 (4.3%)	528 (13.2%)	**<0.001**
Tracheostomy	233 (25.6%)	294 (7.3%)	**<0.001**
Mechanical ventilation duration in days	14383	10160	**<0.001**
Median (IQR)^a^	11 (4-23)	5 (2-11)	
**Endoscopy**	51 (5.6%)	100 (2.5%)	**<0.001**
**Received Sedation**	772 (84.8%)	1382 (34.5%)	**<0.001**
**Imaging radiology **			
**CT**	545 (59.9%)	2457 (61.3%)	0.440
CT Number	1683	6082	
Median (IQR)^a^	3 (2-4)	1 (1-3)	**<0.001**
**MRI**	263 (28.9%)	966 (24.1%)	**0.002**
MRI Number of studies	447	1448	
Median (IQR)^a^	1 (1-2)	1 (1-2)	**0.002**
**Ultrasound**	281 (30.9 %)	827 (20.6 %)	**<0.001**
Ultrasound Number of studies	353	993	
Median (IQR)^a^	1 (1-1)	1 (1-1)	0.103
**Intervention radiology **	187 (20.5%)	643 (16.0%)	**<0.001**
Intervention Number of studies	302	913	
Median (IQR)^a^	1 (1-2)	1 (1-2)	**0.002**
**ECHO**	195 (21.4%)	502 (12.5%)	**<0.001**
ECHO Number of studies Median	218	546	
(IQR)^a^	1 (1-1)	1 (1-1)	0.210
**Antibiotics use**	776 (85.2%)	2759 (68.8%)	**0.001**
One antibiotic	184 (23.7%)	829 (30%)	**<0.001**
Two or more antibiotic	592 (76.3%)	1930 (70%)	
Antibacterial	1702	5749	
Antiviral	106	507	
Antifungal	30	48	
**Blood Transfusion **			
**PRBCs**	139 (15.3%)	206 (5.1%)	**<0.001**
Number PRBCs of Units	325	435	
**FFP**	76 (8.4%)	86 (2.1%)	**<0.001**
Number of FFP Units	340	381	
**Platelets**	67 (7.4%)	75 (1.9%)	**<0.001**
Number of Platelets Units	603	709	
**Cryoprecipitate**	3 (0.3%)	3 (0.1%)	**0.047**
Number of Cryoprecipitate Units	4	8	

^a^ P-value has been calculated using Mann Whiney U-test, bold values represent significant p-value <0.05

**Table 7:** Sub-group analysis comparison of ICU outcome for DNACPR patients and non-DNACPR patients for emergency ICU admission (excluding planned elective admissions for postoperative monitoring)

Variable	DNACPR (*n* = 910)	Non DNACPR (*n* = 4010)	*P*-value
**ICU Length of stay in days (LOS)**	18671	37145	**<0.001**
Mean ± SD^a^	20.5 ± 25.3	9.26 ± 11.9	
**ICU days after DNACPR**	8505	--------	
Mean ± SD^a^	9.37±16.72		
**ICU mortality**	703 (77.3%)	432 (10.8%)	**<0.001**

^a^ P-value has been calculated using Mann Whiney U-test, bold values represent significant p-value <0.05

**Table 8:** Multivariate regression analysis for DNACPR patients utilized resources (sub-group of emergency admissions) after adjustment for severity of illness between groups and organ dysfunction (including APACHI IV, SOFA, & cancer as comorbidity)

Intervention	Adjusted Odds Ratio	95% Confidence Interval	*P* value
Mechanical ventilation	6.63	(5.13–8.57)	**0.0001**
Sedation	8.38	(6.65–10.55)	**0.0001**
CRRT	3.10	(2.49–3.85)	**0.0001**
Tracheostomy	3.16	(2.50- 4.0)	**0.0001**
Ultrasound	1.30	(1.07–1.59)	**0.009**
CT	0.85	(0.71–1.01)	0.060
MRI	1.03	(0.84–1.25)	0.778
Intervention radiology	1.23	(0.99–1.54)	0.064
ECHO	2.04	(1.56–2.66)	**0.0001**
Endoscopy	1.60	(1.00-2.56)	0.050
PRBCs transfusion	2.39	(1.76–3.24)	**0.0001**
Platelet transfusion	2.18	(1.39–3.41)	**0.0001**
Fresh frozen transfusion	2.43	(1.59–3.7)	**0.0001**
Antibiotic	1.05	(0.98–1.12)	0.167

Bold values represent significant p-value <0.05

**Table 9:** Multivariate regression analysis for number of interventions for each category used DNACPR patients (sub-group of emergency admissions) versus Non –DNACPR patients after adjustment for severity of illness between groups and organ dysfunction (including APACHI IV, SOFA, & cancer as comorbidity)

No. of Intervention	Adjusted Odds Ratio	95% Confidence Interval	*P* value
Mechanical ventilation days	4.9	(4.77–5.06)	**0.0001**
CRRT days	2.46	(2.04–2.96)	**0.0001**
Ultrasound	1.20	(1.03–1.35)	**0.021**
CT	1.13	(1.06–1.21)	**0.001**
MRI	1.18	(1.03–1.35)	**0.014**
Intervention radiology	1.27	(1.09–1.50)	**0.003**
ECHO	1.98	(1.58–2.49)	**0.0001**
Endoscopy	1.44	(0.95–2.20)	0.088
PRBCs transfusion	2.35	(1.94–2.85)	**0.0001**
Platelet transfusion	1.7	(1.47–1.98)	**0.0001**
Fresh frozen transfusion	2.31	(1.90–2.80)	**0.0001**

Bold values represent significant p-value <0.05

## Discussion

This research was conducted at one of the largest tertiary care hospitals in Saudi Arabia, examining ICU resource consumption between DNACPR and non- DNACPR patients. In this study 13.9% of ICU admissions were signed DNACPR after ICU admission, and the prevalence of DNACPR order among patients died in ICU was 62% which is comparable to what been reported from other studies in Saudi Arabia between 55 and 66% [11, 29]. This study provides valuable insights into the characteristics, resource utilization, and outcomes of patients with Do Not Attempt Cardiopulmonary Resuscitation (DNACPR) orders admitted to the Intensive Care Unit (ICU). The results from our research are consistent with previous studies, demonstrating that DNACPR patients are more likely to be older and have higher burden of comorbidities, such as diabetes, cardiac disease, chronic renal failure, and chronic liver disease [29–33]. In contrast, cancer comorbidities were prevalent among emergency DNACPR admissions; this finding is consistent with a Turkish study by Topcu et al. in which malignancy was one of the commonest reasons for admissions among DNACPR patients [34]. 

The commonest reasons for ICU were septic shock and respiratory failure, but more prevalent among DNACPR patients were, a trend supported by Wang et al. [33] and Deng et al. [35] among DNACPR patients. Also, Cheng et al. [36] reported that patients with respiratory failure often exhibited high DNACPR rates, indicating a trend toward limiting aggressive treatment in individuals with poor prognoses [36]. Our study found that DNACPR patients had significantly higher APACHE IV and SOFA scores upon admission to the ICU compared to non-DNACPR patients. This finding is consistent with a Korean study by Choi et al. and a recently published study from Saudi Arabia by Asiri et al. [29, 37], which reported higher APACHE II and SOFA scores among DNACPR patients. In contrast, our study used APACHE IV. High APACHE and SOFA scores were strong indicators of illness severity at ICU admission, demanding high resource utilization, and are associated with a poorer prognosis [29, 38, 39].

Furthermore, our study findings indicate that DNACPR patients utilized significantly higher critical care resources following the DNACPR order, including mechanical ventilation, dialysis, endoscopy, radiological imaging, sedation, and blood products. These results align with studies in Saudi Arabia, which reported a high prevalence of mechanical ventilation among DNACPR patients. A recently published study in a pediatric ICU showed that DNACPR patients tend to have a high prevalence of mechanical ventilation and dialysis [12, 29]; however, other interventions were not measured in those studies. Additionally, a close pattern of resource consumption was observed in our study, as reported from study in Taiwan by Yu-chen Huang and Shiu et al. [17, 40], in which Despite the DNACPR decision, that physician practices did not shift toward palliative or comfort care, as evidenced by the continued high usage of ICU resources [40].

Moreover, DNACPR patients had significantly longer ICU stays and higher mortality rates compared to non-DNACPR patients, aligning with a recently published study in a Saudi Arabian adult ICU tertiary center, which reported a mortality rate of 67.8% among DNACPR patients, compared to 8.5% among non-DNACPR patients [41]. Also, the significant long stay and higher mortality among DNACPR patients in our study were consistently reported in previous studies from different countries [22, 40, 42–44].

The reported poor outcome among DNACPR patients in this study, despite the extensive intensive care interventions, is a common pattern in published studies assessing the outcomes of DNACPR patients. This raises concerns regardingg the timing of DNACPR orders before ICU admission. This highlights the need for considering earlier palliative care consultations to ensure optimal resource allocation and provide a more patient-centered approach to care [12, 17, 35, 36, 38–41]. The findings of our study are particularly relevant given the unique socio-cultural and clinical context of DNAR practices in Saudi Arabia. While the official Islamic law (Fatwa) [7–9] on DNAR and the national MOH guideline primarily empowers physicians to make the decisions, including withdrawal of care without the need for family consent, with substantial cultural value placed on family bonding in Saudi Arabia strongly considers family involvement in the patient’s healthcare decisions [5, 6, 45, 46]. On the other hand, families limited knowledge about DNAR and palliative care, may lead to miscommunication that a patient might be exposed to under care if a DNAR decision was agreed upon [45–47]. Balancing these factors is challenging and often results in inconsistencies in practice, particularly when families are not fully informed or disagree with a Do-Not-Attempt resuscitate (DNAR) recommendation. Moreover, there may be shortcomings in individual physician’s knowledge of DNAR legality, specifically around the withdrawal of care [48]. This situation also causes delays in instituting early DNAR decisions, particularly regarding the withdrawal or withholding of care and late DNACPR orders. This practice is consistent with what is reported from Taiwan by Yu-chen Huang and Shiu et al. where significant interventions continued to be used by physicians despite DNAR order, while the practice limited to DNACPR only [40]. In this context, admissions of terminally ill patients to ICU is still not uncommon as reflected in our study by the high APACHE and SOFA score among DNACPR group at the time of ICU admission and is associated with extensive use of ICU interventions despite the paradoxical poor outcome among DNACPR patients could be explained in our study. These findings are consistent with similar findings reported by Alayed [12] from the DNACPR patients among pediatric ICU in Saudi Arabia [12].

Based on the findings of our study, efforts should be directed toward providing early appropriate end-of-life care that prioritizes patient comfort, and proper patient selection before ICU admission, while minimizing the strain on healthcare resources. Early Patient and family education, along with frequent engagement when a disease reaches a terminal stage, is crucial. This strategy will facilitate proper and timely end-of-life care in situations where resuscitation is deemed ineffective, ensuring that medical interventions align with patient-centered goals and ethical resource allocation [18, 42–44, 49].

### Strengths & limitations

This study features a large sample size compared to similar published studies and is distinguished as the first of its kind in Saudi Arabia. It provides insight into the nature of DNACPR and compares it to non-DNACPR cases in terms of ICU interventions and outcomes. However, the study’s limitations include its single-center design and retrospective nature. We also acknowledge the fact that the CPR rates in the patients with DNACPR decisions and their outcomes were not available, which could be explored in future studies. Additionally, records related to disagreements with family members are not available. A cost-effectiveness analysis was not conducted, which is an important area for future research consideration.

## Conclusion

Our findings underscore the crucial need to improve how physicians make decisions when caring for patients with DNACPR orders, especially those who are terminally ill. Implementing early patient and family education, as well as end-of-life care planning, is necessary to provide a more thoughtful and patient-centered approach, ensuring that treatment plans align with the expected outcomes for the patient’s condition.

Our results reveal significant differences in both resource utilization and outcomes for patients with DNACPR orders compared to those without DNACPR orders. This highlights the need for comprehensive guidelines that include more specialized care strategies for terminally ill patients in the intensive care unit. Future research should focus on developing methods to enhance the distribution of healthcare resources between early palliative care and intensive care. This will help us strike a balance between doing what’s ethically right and maintaining sustainable healthcare.

## Data Availability

No datasets were generated or analysed during the current study.
